# Von Willebrand Factor Dynamics in Patients with Aortic Stenosis Undergoing Surgical and Transcatheter Valve Replacement

**DOI:** 10.3390/life14080934

**Published:** 2024-07-25

**Authors:** Andrei Emanuel Grigorescu, Andrei Anghel, Claudia Koch, Florin George Horhat, Delia Savescu, Horea Feier

**Affiliations:** 1 Department of Cardiology, “Victor Babes” University of Medicine and Pharmacy Timisoara, 300041 Timisoara, Romania; grigorescu.andrei@umft.ro (A.E.G.); horea.feier@umft.ro (H.F.); 2 Research Center of the Institute of Cardiovascular and Heart Disease of Timisoara, 300310 Timisoara, Romania; 3 Division of Cardiovascular Surgery, Institute for Cardiovascular Diseases, 300391 Timisoara, Romania; 4 Doctoral School Medicine-Pharmacy, “Victor Babes” University of Medicine and Pharmacy Timisoara, 300041 Timisoara, Romania; 5 Department of Biochemistry, “Victor Babes” University of Medicine and Pharmacy Timisoara, 300041 Timisoara, Romania; 6 Center for Translational Research and Systems Medicine, “Victor Babes” University of Medicine and Pharmacy Timisoara, 300041 Timisoara, Romania; 7 Department of Microbiology, Multidisciplinary Research Center on Antimicrobial Resistance (MULTI-REZ), “Victor Babes” University of Medicine and Pharmacy, 300041 Timisoara, Romania; 8 Department of Laboratory, Children’s Emergency Hospital “Louis Turcanu”, 300001 Timisoara, Romania

**Keywords:** aortic stenosis, aortic valve replacement, von Willebrand factor, hemostasis, surgical aortic valve replacement, transcatheter aortic valve replacement

## Abstract

Aortic stenosis (AS) is a prevalent valvular disorder that poses a significant burden on healthcare systems due to its debilitating symptoms and high mortality rates if left untreated. Surgical aortic valve replacement (SAVR) and transcatheter aortic valve replacement (TAVR) are the primary interventions for severe AS, but perioperative complications such as bleeding remain a concern. Von Willebrand factor (VWF), a crucial player in hemostasis, is known to be altered in AS and may contribute to the hemostatic imbalance observed in these patients. This prospective study aimed to investigate the association between prosthetic valve type, size, and postprocedural VWF levels in patients undergoing aortic valve replacement (AVR) for severe AS. This study involved 39 consecutive patients diagnosed with severe AS who underwent SAVR or TAVR. By elucidating the VWF dynamics associated with different prosthetic valves, this study sought to provide valuable insights into personalized valve selection and perioperative management strategies.

## 1. Introduction

Severe aortic stenosis (AS) represents a significant burden on global healthcare systems due to its increasing prevalence, debilitating symptoms, and high mortality rates if left untreated. Aortic stenosis stands as the predominant valvular disorder in both Europe and North America [1] and serves as the primary indication for surgical intervention in aortic valve pathology. Typically, a diagnosis of aortic stenosis occurs beyond the age of 65, with a prevalence of 4–5% among individuals aged over 65 within the general population.

Surgical aortic valve replacement (SAVR) remains the gold standard in treatment for symptomatic severe AS [2,3,4,5], offering significant improvements in symptoms, quality of life, and long-term survival. Transcatheter aortic valve replacement (TAVR) has emerged as a viable alternative to open-heart surgery, especially for elderly patients with concurrent medical conditions. The selection of the most suitable AVR procedure hinges on multiple factors, such as the patient’s age, overall health, anatomical considerations, and the nature and severity of valve pathology. However, despite advances in surgical techniques and prosthetic valve technology, perioperative complications such as bleeding and thrombosis remain major concerns in these patients.

Von Willebrand factor is a pivotal adhesive and multimeric plasma glycoprotein, with its historical origins dating back to 1924 [6,7]. Synthesized primarily in megakaryocytes and endothelial cells, VWF plays a crucial role in platelet adhesion and blood clotting mechanisms [7,8]. The structural integrity and function of VWF multimers are susceptible to physiological degradation, primarily facilitated by the metalloprotease ADAMTS13 (a disintegrin-like and metalloprotease domain with thrombospondin type I motif). Notably, the susceptibility of VWF multimers to degradation by ADAMTS13 is particularly heightened in individuals with the type 2A form of von Willebrand disease [7]. Alterations in VWF levels and function have been implicated in various cardiovascular diseases, including AS, and may contribute to the hemostatic imbalance observed in these patients [7,9,10,11].

While previous studies have investigated changes in VWF levels in patients with AS, limited data are available regarding the impact of prosthetic valve type and size on postprocedural VWF levels in patients undergoing an AVR procedure [7]. Given the diverse characteristics of available prosthetic valves, including mechanical and biological options with varying sizes, understanding the relationship between prosthetic valve characteristics and postoperative VWF levels is crucial for optimizing patient outcomes [7,12,13,14].

Therefore, this prospective study aims to investigate the association between prosthetic valve type, size, and postprocedural VWF levels in patients undergoing AVR for severe AS. By elucidating the VWF dynamics associated with different prosthetic valves, this study seeks to provide valuable insights into personalized valve selection and perioperative management strategies aimed at minimizing bleeding and thrombotic complications in this high-risk patient population, both in the short term and over long-term follow-up.

## 2. Materials and Methods

### 2.1. Study Design and Patient Population

We conducted a prospective study involving 39 consecutive patients diagnosed with severe aortic stenosis (AS) who underwent surgical (SAVR) or transcatheter (TAVR) aortic valve replacement. Patients were included if they met the following criteria: severe AS confirmed by echocardiography and being suitable candidates for an SAVR/TAVR procedure.

### 2.2. Data Collection

Preoperative data were collected for all patients, including demographic information (age, sex), comorbidities (hypertension, diabetes, coronary artery disease, valvulopathies, aortopathies), echocardiographic parameters (aortic valve area, mean pressure gradient across the aortic valve), and laboratory values (hemoglobin, platelet count, coagulation profile). Intraoperative details, such as the type (biological or mechanical) and size of the prosthetic valve, were meticulously recorded. Those data were compared with postoperative values measured prior to discharge.

### 2.3. Echocardiographic Assessment

The assessment of aortic valve stenosis severity was conducted using transthoracic cardiac ultrasound. Peak velocity, mean pressure gradient, and aortic valve area were measured on admission and prior to discharge. Grading of aortic stenosis into mild, moderate, and severe categories was determined based on established criteria [15,16].

### 2.4. Reference Values of EOA for the Prosthetic Aortic Valves

The effective orifice area (EOA) values for the prosthetic aortic valves that were implanted were obtained from manufacturer’s charts, as well as previously published data, as seen in Table 1 [7,17,18] (Table 1). Body Surface Area (BSA) was assessed using Dubois’ formula and was used to compute the indexed area of the aortic valve before the procedure (SOAi), as well as the indexed orifice area of the implanted prosthesis (EOAi).

### 2.5. Blood Sampling and Analysis

Blood samples were collected preoperatively within 24 h before surgery and on postoperative day 7. Samples were obtained via venipuncture using standardized techniques and processed according to established protocols. Serum levels of Factor VIII, VWF:Ag (Manufacturer: Antibodies-online, Jones Boulevard 321, Limerick, PA, USA) as well as ristocetin-induced VWF: Ag activity (Manufacturer: VWF:RCo-HemosIL Reagents 180 Hartwell Road Bedford, MA, USA) were measured using ELISA techniques

### 2.6. Patient-Prosthesis Mismatch Evaluation

In this study, we employed the model proposed by Pibarot and Rahimtoola to calculate Patient-Prosthesis Mismatch (PPM), a parameter crucial for evaluating the adequacy of prosthetic valve sizing relative to patient body size) [2,7,19]. As described by Rahimtoola and corroborated by Pibarot and colleagues, PPM is delineated more precisely by relating the indexed Effective Orifice Area (EOA) of a prosthesis into categories of severity: mild (indexed EOA > 0.85 cm^2^/m^2^), moderate (indexed EOA between 0.65 and 0.85 cm^2^/m^2^), and severe (indexed EOA < 0.65 cm^2^/m^2^) [2,7,19]. This methodological approach allows for a nuanced assessment of the degree of patient-prosthesis mismatch, enabling a more comprehensive understanding of its impact on postoperative outcomes in patients undergoing aortic valve replacement.

### 2.7. Statistical Analysis

Normality was assessed using Shapiro–Wilk’s test. Continuous variables were presented as mean ± standard deviation, and as median [interquartile range (IQR)]. Categorical variables were presented as frequencies and percentages. To assess the correlation between variables, we employed Spearman’s rank test (Spearman’s Rho). Univariate analysis was conducted by using Student’s *t*-test or Mann–Whitney’s U-test for continuous variables or by a chi-squared test for categorical data. Multivariable analysis was conducted by linear regression or ANOVA to assess for independent variables that effect continuous outcomes. In all cases, a *p* value < 0.05 was considered to assess statistical significance. StataBE version 17.0 was used to conduct statistical analyses (StataCorp LLC, College Station, TX, USA).

### 2.8. Ethical Considerations

This study was conducted in accordance with the principles outlined in the Declaration of Helsinki and approved by the Institutional Ethics Committee of Institute of Cardiovascular Diseases Timisoara (nr.33/09.12.2019). Informed consent was obtained from all participants prior to their inclusion in this study, with approval for their participation and agreement to any future scientific publications.

## 3. Results

### 3.1. Patient Characteristics

A total of 39 consecutive patients undergoing an AVR procedure were included in the study. Most of them were male patients (53.8%) with a mean age of 68.33 ± 9.1 years (range 46–83). They all exhibited severe aortic stenosis, with an aortic orifice area of 0.81 ± 0.15 cm^2^ (indexed aortic orifice area of 0.43 ± 0.08 cm^2^/m^2^ BSA) and a mean gradient of 51.45 ± 13.12 mm Hg. The aortic annulus was 2.28 ± 0.21 cm (range 1.9–2.96 cm), and the ejection fraction was 48.66 ± 8% (Table 2 and Table 3).

Our results did not reveal any significant correlations between these factors and changes in VWF levels throughout the perioperative period. Specifically, we found that age, gender, and ethnicity showed no substantial association with VWF level fluctuations. Similarly, common comorbidities frequently observed in our patient population, including smoking, hypertension, diabetes mellitus, chronic pulmonary disease (CPD), extracardiac arteriopathy (ECA), and renal function impairment did not appear to significantly affect VWF dynamics.

### 3.2. Intraprocedural Aspects

The mean prosthesis size in the TAVR group was 31.12 mm, while in the SAVR group, it was 22.35 mm. For SAVR procedures, the mean cardiopulmonary bypass (CPB) time was 103.13 min [IQR 78–110.5], and the mean aortic cross-clamp time was 62.35 min [IQR 45–78.5]. The length of stay in the intensive care unit (ICU) was markedly different between the two groups, with SAVR patients spending a mean of 4 days, while TAVR patients had a significantly shorter ICU stay with a mean of 0.75 days (Table 4).

Isolated SAVR was performed in 79.49% of patients (n = 31), via a conventional or mini-sternotomy approach, while 20.51% of patients benefitted from a TAVR procedure (n = 8) using a self-expandable endoprosthesis. Associated coronary artery bypass surgery (CABG) was performed in 10.26% of cases (n = 4), while an associated mitral valve procedure, with or without CABG, was undertaken in 10% (n = 4) of patients. One patient benefitted from SAVR and ascending aortic replacement (2.56%).

Bioprosthetic valves were the preferred choice in 58.97% (n = 23) of cases, with mechanical valves utilized in the remaining 41.03% (n = 16). The most commonly used valve model was the Carbomedics Top Hat, accounting for 41.03% (n = 16) of cases, followed by Edwards Lifesciences CE Perimount in 25.64% (n = 10) of cases, while the Medtronic Evolut R valve model was used in 20.51% (n = 8) of cases.

The EOA of the implanted protheses was 1.67 cm^2^ [IQR: 1.5–1.82], with the indexed EOA 0.88 cm^2^ [IQR: 0.73–0.99].

In our study, the decision between TAVR and SAVR was based on an evaluation of patient characteristics, with EUROSCORE II serving as one of the considered factors. The mean EUROSCORE II for TAVR patients was 2.28 (±1.45), compared to 1.98 (±1.79) for SAVR patients. Notably, only one TAVR patient had a EUROSCORE II exceeding 4, while two SAVR patients surpassed this threshold, both of whom underwent associated procedures.

Age was the most significant factor in procedure selection (Table 5). TAVR patients had a mean age of 74 years (±6.67), considerably higher than the SAVR group at 66.8 years (±9.15). This age disparity reflects our clinical approach, where advanced age often favored TAVR. Moreover, the overall clinical condition of the patient played a crucial role in decision making. TAVR was preferentially chosen for patients in poorer condition, even when their clinical profile might have permitted SAVR, aligning with the less-invasive nature of the transcatheter approach.

While few patients in our cohort fell within the 4–8% EUROSCORE II range, several key factors generally influence the choice between TAVR and SAVR in this risk category. These include age, clinical frailty assessment, comorbidities, aortic valve anatomy, presence of coronary artery disease, history of previous cardiac surgery, estimated life expectancy, and patient preference. The final decision for each patient was made considering these factors in conjunction with EUROSCORE II, to tailor the treatment strategy to individual patient needs and risk profiles.

This multifactorial approach to procedure selection underscores the complexity of clinical decision making in aortic valve replacement. It highlights that while risk scores like EUROSCORE II provide valuable information, they should be interpreted within the broader context of each patient’s unique clinical picture.

### 3.3. Von Willebrand Factor Dynamics

**Figure 1:**
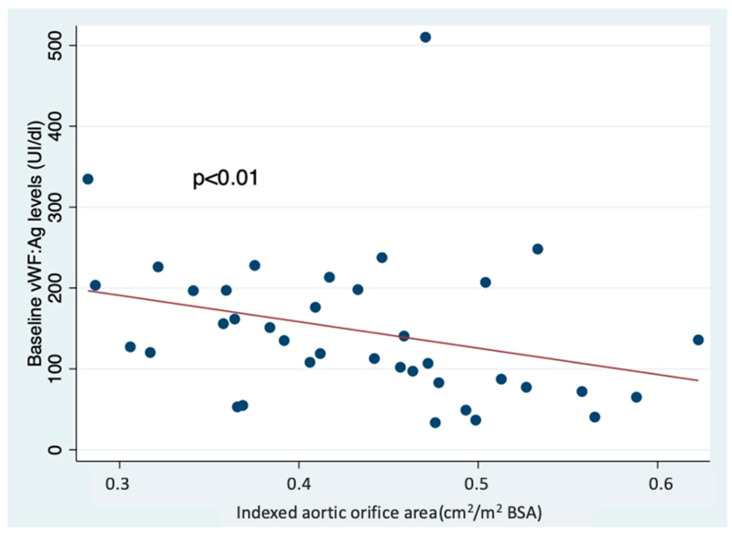
Relationship between preoperative VWF antigen levels and indexed aortic valve area.

The mean von Willebrand factor antigen on admission (VWF:Ag) level was 145.94 ± 91.87 UI/dL [IQR: 82.9–198]. Four patients had a preoperative VWF:Ag level <50 UI/dL. Preoperative von Willebrand antigen levels were inversely correlated with the indexed aortic valve surface area (rho = −0.41, *p* < 0.01) (Figure 1). After the procedure, the values of von Willebrand factor antigen increased to 289.26 UI/dL [IQR: 157.7–345.4] (*p* < 0.01), and no such correlation was found with the indexed orifice area of the implanted prosthesis (rho = −0.22, *p* = −0.16). Furthermore, all four patients who had, at baseline, VWF:Ag < 50 UI/dL increased their plasmatic level to >80 UI/dL after having their aortic pathology treated.

The VWF activity had a similar variation, increasing from 80.34% [IQR: 47.8–122] at baseline to 191.20% [IQR: 118.1–135.4] after the valve replacement procedure (*p* < 0.01).

On the other hand, there were no differences in factor VIII levels in our sample (89.02 UI/dL [IQR: 58.6–103.8] vs. 98.78 UI/dL [IQR: 78.4–111.2], *p* = 0.21), as well as no difference in VWF:Ag/VWF:Activity ratio (2.27 [IQR: 1.27–2.57] vs. 1.95 [IQR: 1.05–2.40], *p* = 0.33).

The difference between preprocedural and postprocedural VWF:Ag levels was 143.32 IU/dL [IQR: 24.4–204.8]. The postprocedural increase in VWF:Ag levels was significantly lower in patients implanted with a percutaneous aortic valve than in patients with surgically implanted ones (2.59 [IQR: −52.5–84.75] vs. 179.63 [IQR: 65.4–233.9], *p* < 0.01). Multivariable analysis with the increase in preoperative VWF:Ag levels (in percentages of the initial value) as the dependent variable revealed that percutaneous aortic valve implantation was associated with the lowest increase (Table 6).

### 3.4. Postoperative Bleeding

In SAVR patients, bleeding was 377.69 mL [IQR: 230–450] within the first 24 h after the procedure, and the baseline value of the vWF:Ag was an independent negative predictor of postoperative drainage (Regression coefficient −1.12, 95% CI = −2.09–−0.14, *p* = 0.02, Table 7).

### 3.5. Influence of PPM

PPM, defined as an indexed effective orifice area <0.85 cm^2^/m^2^ BSA of the implanted prosthesis, was present after the procedure in 48.72% of patients (n = 19). It did not influence the postoperative VWF:Ag levels (285.43 UI/dL [IQR: 135.65–382.9] vs. 293.30 [IQR: 222.9–345.4], *p* = 0.88), VWF activity (178.33 [IQR: 119.2–130.9] vs. 204.76 [IQR: 115.8–399.2], *p* = 0.56) or factor VIII levels (100.38 UI/dL [IQR: 75.3–111.5] vs. 97.10 UI/dL [IQR: 80.4–111.2], *p* = 0.79).

### 3.6. Blood Group

At baseline, patients with blood group 0 had lower vWF activity levels than those who exhibited a blood group antigen (1.18 ± 0.53 vs. 2.59 ± 1.92, *p* = 0.03), but the serum VWF:Ag levels were similar (100.98 UI/dL [IQR: 72–118.9] vs. 159.43 UI/dL [IQR: 87.3–207], *p* = 0.09), as were the factor VIII levels (77.33 UI/dL [IQR: 57.4–88.4] vs. 92.52 UI/dL [IQR: 59.6–105.4], *p* = 0.42). Those differences disappeared after the valve replacement procedure in the first week (VWF activity: 2.25 [IQR: 1.17–2.40] vs. 1.86 [IQR: 1.05–2.33], *p* = 0.48; VWF:Ag: 364.8 UI/dL [IQR: 180.6–447.4] vs. 266.60 UI/dL [IQR: 157.7–318], *p* = 0.13; fVIII levels 92.28 UI/dL [IQR: 73.2–89] vs. 100.73 UI/dL [IQR: 81.4–113.2], *p* = 0.56).

## 4. Discussion

**Figure 2:**
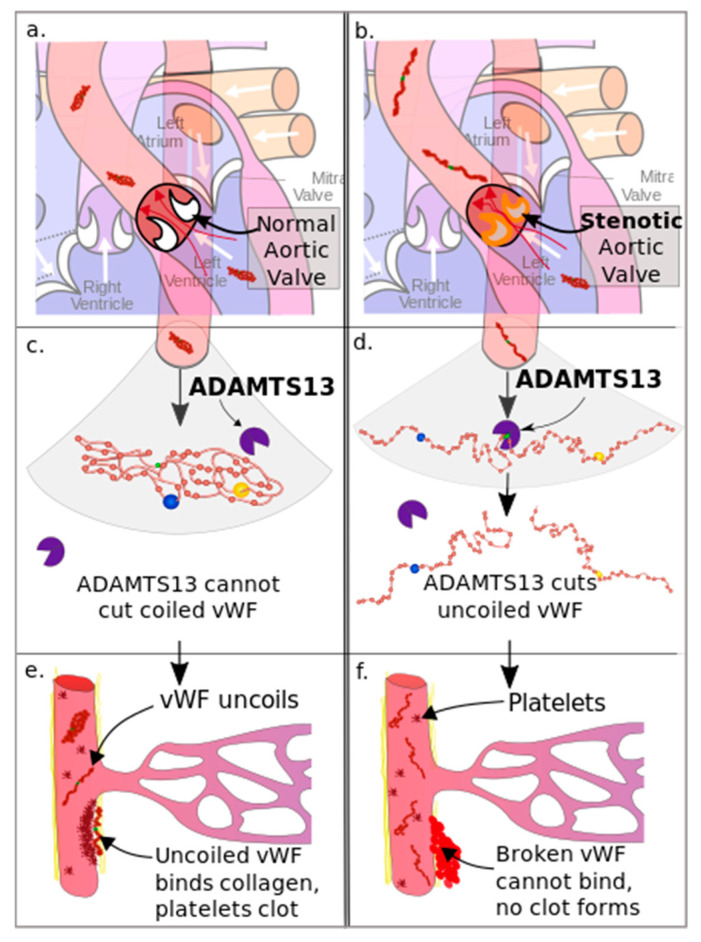
The degradation of VWF due to AS [23]. (**a**) Nomral aortic valve, (**b**) Stenotic aortic valve, (**c**) ADAMTS13 cannot cut coiled VWF, (**d**) ADAMTS13 cuts uncoiled VWF, (**e**) VWF uncoils and binds collagen platelets clot, (**f**) Broken VWF cannot bind and doesn’t forms clot.

Acquired von Willebrand factor deficiency, notably type 2A of von Willebrand syndrome, has been documented in individuals diagnosed with aortic valve stenosis. This phenomenon is attributed to the elevated shear forces experienced during the passage of blood through the narrowed valve orifice. These heightened shear forces induce structural alterations in the von Willebrand factor molecule, exposing certain amino acid bonds and leading to the proteolysis of the highest-molecular-weight multimers (HMWM) of VWF, which are critical for platelet-mediated hemostasis(Figure 2) [7,20]. Consequently, a reduction in the levels of circulating HMWM of VWF, particularly below 10.5%, escalates the risk of bleeding events [7,21]. Frank and coworkers, as well as other studies, have reported that patients diagnosed with severe aortic stenosis exhibit HMWM of VWF levels up to 50% lower than the normal range [7,9].

The severity of acquired VWF deficiency correlates with the degree of aortic stenosis. Studies have shown that patients with severe aortic stenosis may have levels of HMWM VWF up to 50% lower than normal values [9]. The severity of acquired von Willebrand disease (VWD) appears to be strongly related to the pressure gradient across the aortic valve [22]. This finding underscores the critical role of high shear stresses in the proteolysis of HMW multimers of VWF.

Structural irregularities within the von Willebrand factor have been observed to exhibit a direct association with the trans-prosthetic pressure gradient. Consequently, individuals who develop patient-prosthesis mismatch following aortic valve replacement may experience persistent high shear forces, despite the prosthesis functioning normally. This scenario could potentially contribute to the development of acquired VWF deficiency even after a valve replacement procedure [7].

The primary aim of our study was to investigate the impact of different aortic valve replacement techniques on von Willebrand factor dynamics and postoperative outcomes in patients with severe aortic stenosis. Our results provide insights into the hemostatic alterations following surgical aortic valve replacement and transcatheter aortic valve replacement and their implications on patient management.

### 4.1. VWF Dynamics

Our study demonstrated a significant increase in VWF antigen levels postoperatively, highlighting a restoration of hemostatic function following AVR. The mean preoperative VWF level was low at 145.94 UI/dL [IQR: 82.9–198], reflecting the altered VWF levels due to AS. Postoperatively, VWF levels nearly doubled to 289.26 UI/dL [IQR: 157.7–345.4], indicating a substantial recovery. This finding is consistent with previous studies showing that the removal of the stenotic valve alleviates high shear stress, thereby reducing VWF degradation [7,9,12,13].

While not the focus of our current study, it is worth noting for future research that prolonged cardiopulmonary bypass (CPB) time, especially in combined surgical procedures, can introduce additional complexities to the post-operative hemostatic environment. The interplay between CPB-related effects and VWF dynamics represents an important area for future investigation. Subsequent studies would provide a more complete picture of the multifaceted nature of hemostasis management.

The increase in VWF levels was significantly lower in patients undergoing TAVR compared to SAVR. This could be attributed to the less invasive nature of TAVR, which might induce lower levels of systemic inflammation and hemostatic activation compared to SAVR [24]. The lower short-term increase in VWF levels observed in TAVR patients, compared to those undergoing SAVR, suggests that the hemostatic recovery process may differ initially between the two procedures. However, this study’s short follow-up period limits our understanding of the long-term normalization of VWF levels. Future research should focus on long-term evaluations to determine the full trajectory of hemostatic recovery following TAVR.

### 4.2. Impact of Patient-Prosthesis Mismatch

In our study, we utilized a range of prosthetic valves tailored to individual patient needs. For surgical aortic valve replacement (SAVR), we employed both bioprosthetic and mechanical valves. The bioprosthetic options included the Hancock II (Medtronic, Minneapolis, MN, USA), in sizes of 23 mm and 25 mm, and the Edwards Perimount (Edwards Lifesciences, Irvine, CA, USA), ranging from 19 mm to 25 mm. The Hancock II is a porcine valve with a reported effective orifice area (EOA) of 1.3 cm^2^ for the 23 mm size and 1.5 cm^2^ for the 25 mm size. The Edwards Perimount, a bovine pericardial valve, offers EOAs of 1.1 cm^2^, 1.3 cm^2^, 1.5 cm^2^, and 1.8 cm^2^ for sizes 19 mm, 21 mm, 23 mm, and 25 mm, respectively. For mechanical valve implantations, we used the Carbomedics Standard and Top Hat models (LivaNova, London, UK) in sizes ranging from 21 mm to 25 mm. These valves provide EOAs of 1.5 cm^2^, 1.7 cm^2^, 2.0 cm^2^, and 2.3 cm^2^ for the 21 mm, 23 mm, 25 mm, and 27 mm sizes, respectively. In transcatheter aortic valve replacement (TAVR) procedures, we utilized the Medtronic Evolut R (Medtronic, Minneapolis, MN, USA) with sizes ranging from 26 mm to 34 mm. The Evolut R system offers EOAs of 1.9 cm^2^, 2.1 cm^2^, 2.3 cm^2^, and 2.5 cm^2^ for the 23 mm, 26 mm, 29 mm, and 34 mm valve sizes, respectively.

The high incidence of patient-prosthesis mismatch (PPM) in our study, affecting nearly half of the patients who underwent aortic valve replacement, can be attributed to a combination of anatomical and anthropometric factors. Our SAVR cohort presented with a mean height of 1.67 ± 0.08 m, a mean weight of 82.25 ± 20.9 kg, and a resultant body surface area (BSA) of 1.90 ± 0.24 m^2^. Notably, these patients had a mean aortic annulus diameter of 2.25 ± 0.20 cm, which is relatively small in proportion to their BSA (Table 8). This mismatch between a small native aortic annulus and a larger body size significantly contributes to the challenge of achieving optimal prosthesis sizing. In clinical practice, the decision-making process involves carefully balancing the risk of PPM against the potential complications associated with more extensive procedures, such as aortic root enlargement. These additional procedures can prolong surgery time and potentially increase perioperative risks. While we consistently strive to minimize PPM, it is important to acknowledge that factors including patient anatomy, surgical considerations, and the range of available prosthesis sizes can sometimes limit our ability to completely avoid PPM, particularly in complex cases. Our approach prioritizes overall patient safety and clinical benefit, occasionally accepting a degree of PPM when the alternatives are deemed to pose greater risks to the patient.

Contrary to our hypothesis, PPM did not affect postoperative VWF levels, VWF activity, or factor VIII levels. Although several publications [7,12,13,14] suggest that PPM influences VWF levels, our study did not support this hypothesis, likely due to the short-term nature of our analysis. This finding suggests that the immediate hemostatic benefits of AVR might not be compromised by the presence of PPM in the short term. However, the long-term implications of PPM on VWF levels and clinical outcomes remain to be elucidated and require extended follow-up studies.

### 4.3. Influence of Blood Group on VWF Levels

We observed baseline differences in VWF activity levels between patients with blood group 0 and those with other blood groups, consistent with existing literature indicating lower VWF levels in blood group 0 individuals [25,26,27]. Postoperatively, these differences disappeared, underscoring the uniform hemostatic recovery following AVR regardless of blood group. This finding supports the notion that the hemostatic improvements conferred by AVR are robust across different patient subgroups.

### 4.4. Postoperative Bleeding

Our study identified baseline VWF levels as an independent negative predictor of postoperative bleeding in SAVR patients. Higher preoperative VWF levels were associated with reduced postoperative bleeding, emphasizing the protective role of adequate VWF levels in mitigating bleeding risks. This finding underscores the importance of preoperative hemostatic evaluation and optimization in patients undergoing SAVR to enhance postoperative outcomes.

## 5. Limitations

One notable limitation of our study is the short-term follow-up period, which restricts our ability to draw conclusions about the long-term hemostatic function and clinical outcomes post-AVR. Additionally, the relatively small sample size may limit the generalizability of our findings. Future studies with larger cohorts and extended follow-up periods are necessary to validate our results and explore the long-term implications of AVR on the dynamics of VWF levels.

## 6. Conclusions

Our study highlights the significant VWF level improvements following AVR in patients with severe AS, with distinct differences observed between SAVR and TAVR techniques. The normalization of VWF levels postoperatively underscores the efficacy of AVR in mitigating hemostatic impairment associated with AS. However, the lower increase in VWF levels following TAVR and the lack of impact of PPM on short-term VWF dynamics warrant further investigation. Preoperative hemostatic assessment, particularly VWF evaluation, could serve as a valuable tool in predicting and managing postoperative bleeding risks. Future research should focus on long-term outcomes and the potential role of PPM in influencing hemostatic function over time.

## Figures and Tables

**Table 1:** Normal reference values of effective orifice areas for the prosthetic aortic valves. Adapted from [7,17,18].

Prosthetic Valve Size(mm)	19	21	23	25	27	29
Bioprosthetic valves						
Hancock II	−	1.2 ± 0.2	1.3 ± 0.2	1.5 ± 0.2	1.6 ± 0.2	1.6 ± 0.2
Carpentier-Edwards Perimount	1.1 ± 0.3	1.3 ± 0.3	1.5 ± 0.4	1.8 ± 0.4	2.1 ± 0.4	2.2 ± 0.4
Biocor (Epic)	1.0 ± 0.3	1.3 ± 0.5	1.4 ± 0.5	1.9 ± 0.7	-	-
Mechanical valves						
Carbomedics Standard and Top Hat	1.0 ± 0.4	1.5 ± 0.3	1.7 ± 0.3	2.0 ± 0.4	2.5 ± 0.4	2.6 ± 0.4
**TAVR Prosthesis**	**23**	**26**	**29**	**31**	**34**	
Medtronic Evolut R	1.66 ± 0.42	1.82 ± 0.43	1.98 ± 0.56	1.98 ± 0.59	2.56 ± 0.77	

**Table 2:** Echographic assessment.

Variable	Mean	Min	Max
Ao. Anulus (cm)	2.28	1.9	2.96
Pmax (mmHg)	79.76	16	134
Pmed (mmHg)	51.45	34	90
Valve area (cm^2^)	0.81	0.55	1.2
Indexed valve area (cm^2^)	0.43	0.28	0.62
EF (%)	48	25	55
VTD (mL)	110.48	70	215

**Table 3:** Baseline clinical characteristics on admission.

	Mean		Median		Q1		Q3	
	SAVR	TAVR	SAVR	TAVR	SAVR	TAVR	SAVR	TAVR
**Age**	66.87	74	70	76	64	70	73	77.5
**Height (m)**	1.67	1.67	1.66	1.7	1.6	1.62	1.75	1.72
**Weight (kg)**	82.22	82	85	82.5	62	70	96	92.5
**BMI (kg/m^2^)**	29.37	29.64	27.16	27.86	23.50	25.19	33.79	33.55
**BSA (m^2)^**	1.90	1.90	1.95	1.88	1.69	1.81	2.10	2.03
**Hemoglobin (g/dL)**	13.7	12.51	13.7	13.5	12.8	10.65	14.8	14.3
**Platelets (/mm^3^)**	208,709	194,125	201,000	179,000	174,000	153,000	234,000	231,000
**VWF Antigen (UI/dL)**	131.37	202.4	120.2	166.25	77.3	97.85	198	265.9
**Factor VIII (UI/dL)**	95.30	64.67	87.6	59.9	61.8	44	105.4	77.4
**VWF Activity (%)**	79.25	84.56	81.5	95.05	45.9	51.7	122	121.8
**EOA (cm^2^)**	1.52	2.25	1.5	2.27	1.3	1.98	1.7	2.56
**iEOA (cm^2^)**	0.79	1.16	0.77	1.18	0.71	1.00	0.92	1.29
**Aortic anulus (cm)**	2.25	2.40	2.2	2.35	2.1	2.25	2.4	2.45
**EF (%)**	48.48	49.375	50	52.5	45	50	55	55
**Vmax (m/sec)**	4.50	4.38	4.4	4.31	4.17	4.05	4.85	4.7
**Pmax (mmHg)**	80.35	77.5	77	72.5	66	66.5	94	87.5
**Pmed (mmHg)**	52.24	48.62	47	47	41	41.5	60	56.5
**Scont (cm^2^)**	0.81	0.82	0.8	0.8	0.7	0.72	0.9	0.9
**Scont indexed (cm^2^)**	0.43	0.43	0.42	0.45	0.36	0.35	0.49	0.48
**PASP (mmHg)**	42	46.25	40	40	35	40	45	50
**Bicuspidy %**	19.35	12.5						
**Hypertension %**	67.74	100						
**Diabetes %**	35.48	50						
**Smoking %**	9.68	12.5						
**CPD %**	12.9	12.5						
**ECA %**	3.23	25						
**Creatinine (mg/dL)**	0.91	1.21	0.82	1.08	0.71	0.93	1.11	1.41
**Creatinine clearance (mL/min)**	92.09	63	81	52.5	61	43	112	90.5
**EUROSCORE II (%)**	1.98	2.28	1.56	2.11	1.14	1.06	1.86	2.88

**Table 4:** Intraprocedural and postprocedural outcomes.

	Mean		Median		Q1		Q3	
	SAVR	TAVR	SAVR	TAVR	SAVR	TAVR	SAVR	TAVR
**Prosthesis size**	22.35	31.12	23	31.5	21	29	23	34
**CBP time (min)**	103.13		94.5		78		110.5	
**Cross-clamp time (min)**	62.35		57		45		78.5	
**Drainage (mL)**	377.69		310		230		450	
**Days ICU**	4	0.75	2	0	1	0	3	0.5
**Days post procedure**	5.96	4.25	6	4.5	5	3	7	5

**Table 5:** TAVR vs. SAVR EUROSCORE II.

Variable	TAVR	SAVR
**Euroscore2 (mean)**	2.28 (±1.45)	1.98 (±1.79)
**Age (mean)**	74 (±6.67)	66.8 (±9.15)

**Table 6:** Multivariable analysis of variables affecting the increase in VWF:Ag levels.

Variable	Coefficient	95% CI	*p*
Ejection fraction	6.53	−0.44–13.51	0.06
Indexed postoperative prosthetic orifice area	2.07	−2.02–6.18	0.31
TAVR procedure	−2.49	−4.54–−0.44	0.01

**Table 7:** Multivariable analysis of variables affecting bleeding in surgical patients.

Variable	Coefficient	95% CI	*p*
Preoperative VWF:Ag levels	−1.12	−2.09–−0.14	0.02
Preoperative fVIII levels	0.80	−0.38–1.98	0.17
Preoperative anticoagulant treatment	4.67	−129.3–138.6	0.94

Note: Anticoagulant treatment refers to use of antivitamins K, NOACs, or antiaggregant medication.

**Table 8:** SAVR patients’ characteristics.

Variable	SAVR
**Height (mean)**	1.67 (±0.08)
**Weight (mean)**	82.25 (±20.9)
**BSA (m^2^)**	1.90 (±0.24)
**Aortic anulus (cm)**	2.25 (±0.20)

## Data Availability

The original contributions presented in the study are included in the article, further inquiries can be directed to the corresponding author.
